# Supplementary material for: WH2 and proline‐rich domains of WASP‐family proteins collaborate to accelerate actin filament elongation
Source: EMBO J. 2017 Nov 15;37(1):102–21. doi: 10.15252/embj.201797039 (PMC5753033; doi:10.15252/embj.201797039)
Supplement: Supplementary file 1 — Expanded View Figures PDF [file EMBJ-37-102-s001.pdf]

Expanded View Figures

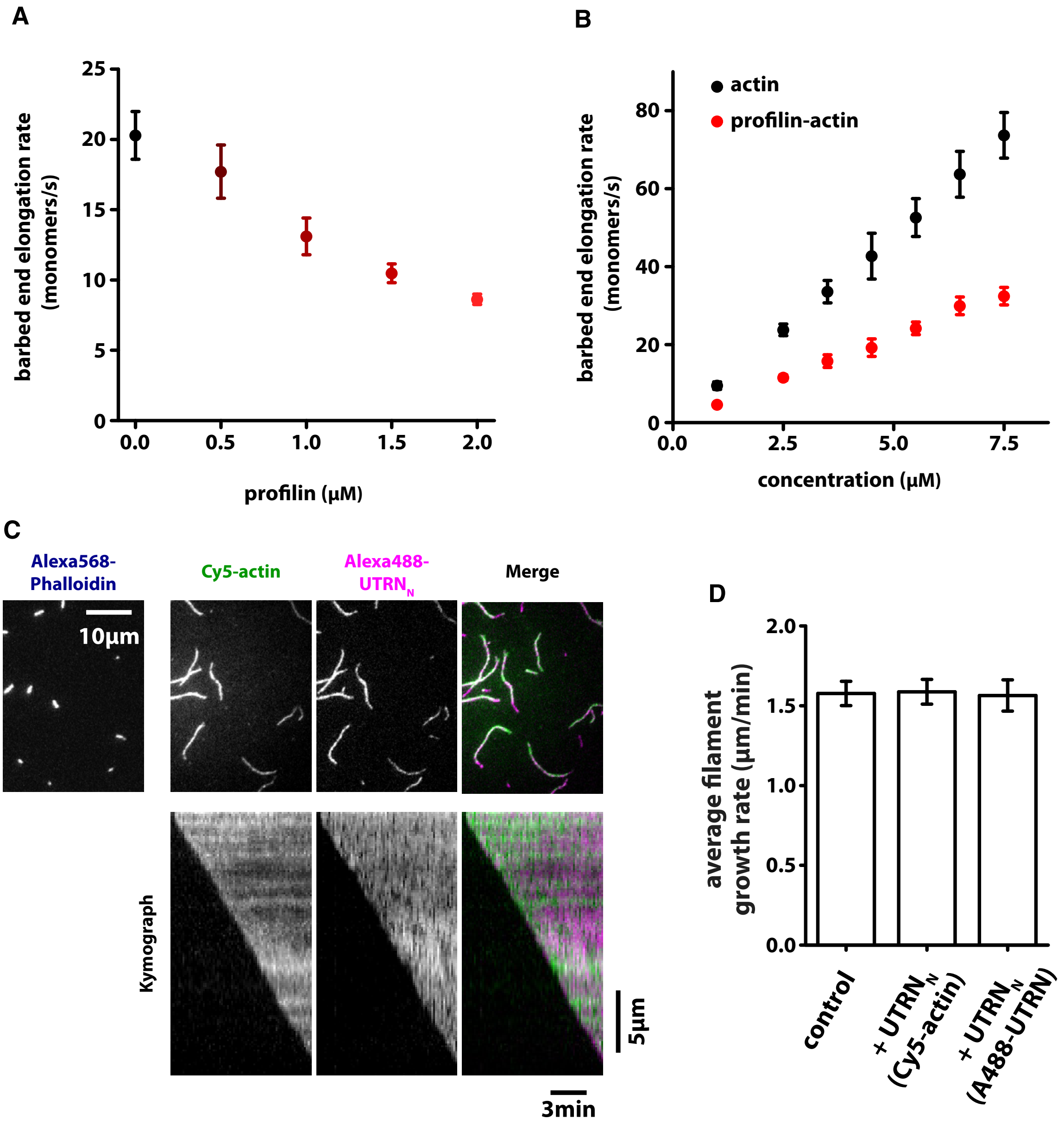

Figure EV1. Effect of profilin or an utrophin-based probe on actin elongation as measured by TIRF-M.

A Barbed end elongation rates determined from time-lapse TIRF microscopy of single-filament growth from 2 µM actin (30% Cy5 labeled) in the presence of indicated profilin concentrations. At least 40 filaments were analyzed per condition.

B Barbed end elongation rates determined from time-lapse TIRF microscopy of single-filament growth at indicated actin (black) or profilin–actin (red) concentrations. At least 40 filaments were analyzed per condition.

C Snapshot images (top panels) or kymographs (bottom panels) from time-lapse TIRF microscopy of single-filament growth from Alexa568 phalloidin-stabilized seeds in the presence of 1 µM actin (30% Cy5 labeled) in the presence of 5 nM Alexa488-UTRN$_N$.

D Barbed end polymerization rates for either a control sample containing only 1 µM of actin alone (left bar) or with the addition of 5 nM Alexa488-UTRN$_N$ either visualized using labeled actin (middle bar) or labeled UTRN$_N$ (right bar). Note that the presence of the filament-binding probe at this very low concentration does not affect actin polymerization. Also, the growth velocities determined from either actin or UTRN$_N$ channel were indistinguishable, further demonstrating the validity of the probe. At least 40 filaments were analyzed per condition.

Data information: All error bars are SD.

© 2017 The Authors

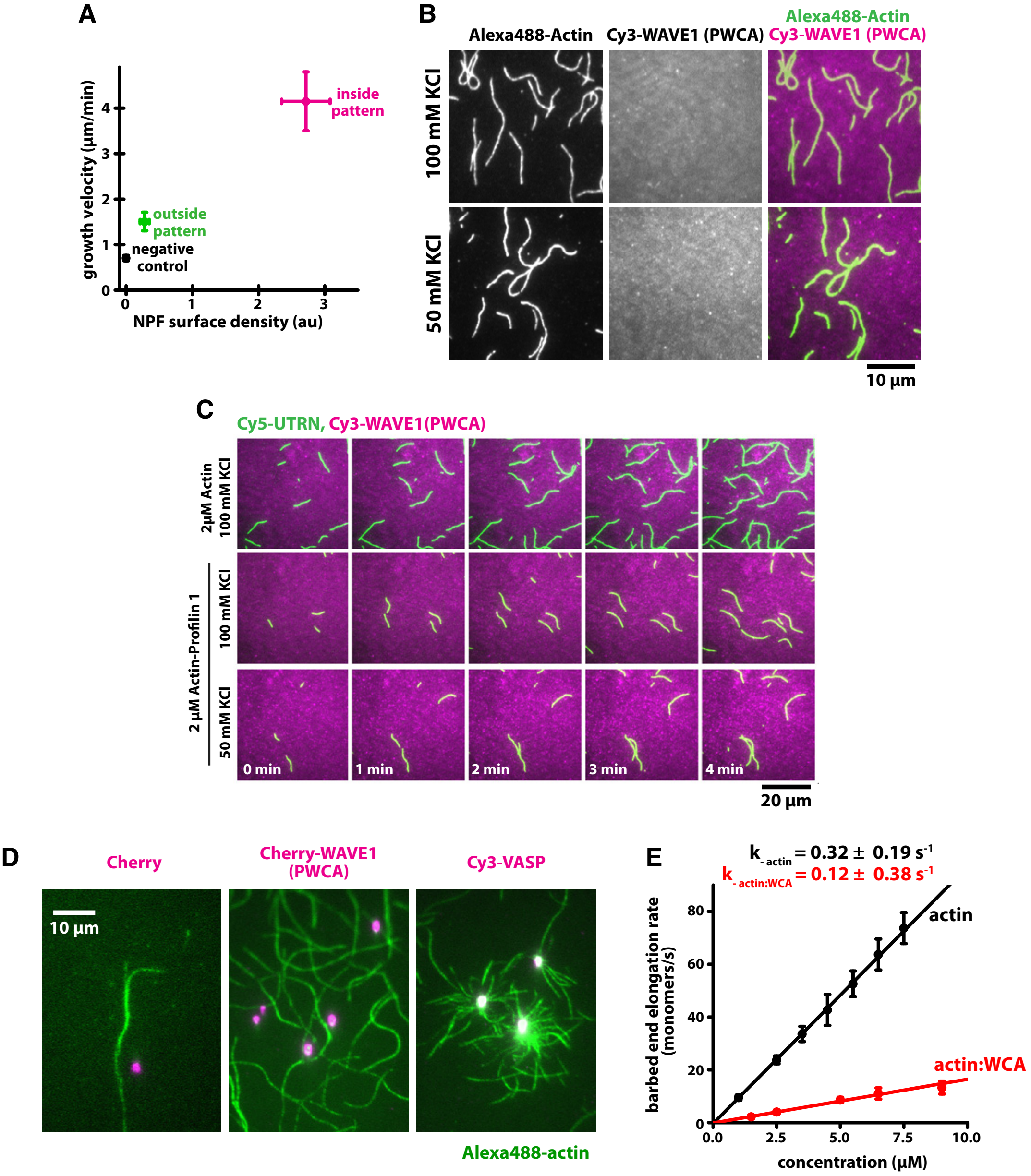

Figure EV2. WAVE1 does not processively bind to filament barbed ends as individual molecules or when clustered on a surface.

A Barbed end growth velocities in 1 µM profilin–actin as a function of measured mCherry-WAVE1 surface density either outside (green) or inside (magenta) of an NPF patch or a non-WAVE1-treated coverslip that was used as an independent control (black) as in Fig 2A–E. Note that the passivated region outside of the high-density patch still contains about 10% residual surface-bound WAVE1. Error bars are SD. At least 40 filaments were analyzed per condition.

B Soluble Cy3-PWCA (0.5 µM) does not associate with the sides or barbed ends of phalloidin-stabilized actin filaments (30% Alexa488-labeled actin plus dark phalloidin). Actin filament-binding was characterized in the presence of both 50 mM and 100 mM KCl.

C Soluble Cy3-PWCA (0.5 µM) does not associate with the sides or barbed ends of dynamically elongating actin filaments. Filaments were polymerized in the presence of either 2 µM actin or 2 µM actin–profilin (50 and 100 mM KCl). Actin filaments were visualized using soluble Cy5-UTRN actin-binding domain (5 nM).

D TIRF microscopic images of streptavidin-functionalized polystyrene microspheres to which biotinylated version of the indicated proteins (magenta) were immobilized about 5 min after immersion in 1 µM actin (containing 10% Alexa488-actin, green). Note that VASP-coupled beads spawn filaments which are persistently attached through their elongating barbed ends, while control and WAVE1 (PWCA) beads to not bind to filaments.

E Barbed end elongation rates (dots) determined from time-lapse TIRF microscopy of single-filament growth at indicated actin (black) or WAVE1(WCA)-actin (red) concentrations. Actin data are the same as in Fig EV1B). Off-rates of actin or WAVE1(WCA)-actin from filament ends were determined from the y-intercept of linear fit to the data (lines). Error bars are SD. At least 40 filaments were analyzed per condition.

 © 2017 The Authors

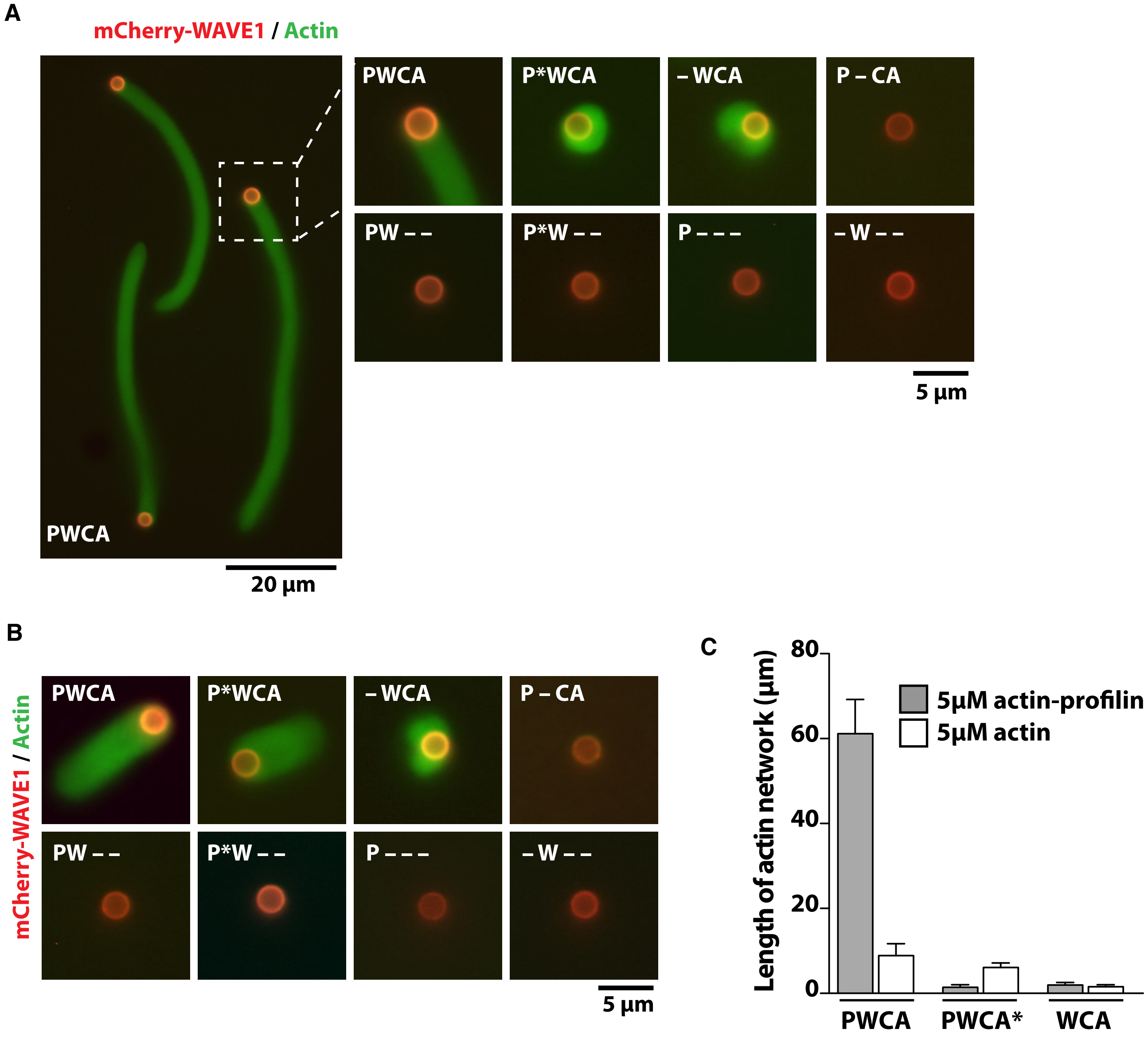

Figure EV3. Individual WAVE1 domains cannot activate the Arp2/3 complex or generate dendritic networks.

A, B The presence of a functional PRD is required for efficient actin nucleation and filament elongation in the presence of actin–profilin. Representative wide-field fluorescence images of dendritic actin networks (Cy5-Actin, green) assembled from lipid-coated beads to which indicated mCherry-WAVE1 (red) constructs in the presence (A) 5 µM actin (5% Cy5-labeled) or (B) 5 µM profilin–actin. Reaction conditions include LCBs (96% DOPC, 4% Ni-NTA lipids), 50 nM Arp2/3, and 200 nM CP. Reaction was quenched with 5-fold molar excess Latrunculin B and phalloidin after 10 min of actin network assembly.

C Average actin network growth rate measured for $n > 50$ actin comet tails per condition. Errors bars represent SD.

© 2017 The Authors

Figure EV4. The WAVE1 PRD domain contains six proline-rich sites which interact with profilin with distinct affinities as determined by analytical ultracentrifugation.

A Primary sequence of the wt WAVE1 PRD (top) compared to single poly-proline site mutants (bottom) as indicated.

B Molecular weight of indicated WAVE1 PRD variants measured by sedimentation equilibrium ultracentrifugation. Equilibrium radial distributions at 7 K, 10 K, and 14 K rpm are shown.

C Interaction between WAVE1 PRD[B] and profilin measured by sedimentation equilibrium ultracentrifugation. Equilibrium radial distributions of WAVE1 PRD[B] in the presence of 91.2, 44.8, and 21.7 μM profilin at 7 K, 10 K, and 14 K rpm are shown. Equilibrium traces are fit using a monomer–dimer equilibrium model (see Materials and Methods).

D WAVE1 PRD contains six proline-rich sites that each interact with profilin 1 and profilin 2 with distinct affinities as determined by analytical ultracentrifugation. Affinities for profilin 1 or profilin 2 binding for either the profilin-binding deficient PRD mutant (PRD null) or indicated single site constructs with individual binding sites re-introduced. The residual interaction between profilin and PRD null mutant was modeled as a 1:1 interaction with a K_D of 138 μM. We consider this low-affinity interaction as non-specific. All other data with WAVE1 PRD single binding site variants were analyzed using a two-site, independent binding model with one site fixed to the measured non-specific affinities.

 © 2017 The Authors

A

```
                                A                                                B                          C                 D
human WAVE1 277------------EPPPPPPMHGAGDAKPIPTCISSATGLIENRPQSPATGRTPVFVSPTPPPPPPPLPSALSTSSLRASMTSTPPPPVPPPPPPPATALQAPAVPPPPAPLQ-376
      PRD[null]            EPGSGSPMHGAGDAKPIPTSISSATGLIENRPQSPATGRTPVFVSPTPGSGSPPLPSALSTSSLRASMTSTGSGSVPGSGSPPATALQAPAVGSGSAPLQ-
      PRD[A]               EPPPPPPMHGAGDAKPIPTSISSATGLIENRPQSPATGRTPVFVSPTPGSGSPPLPSALSTSSLRASMTSTGSGSVPGSGSPPATALQAPAVGSGSAPLQ
      PRD[B]               EPGSGSPMHGAGDAKPIPTSISSATGLIENRPQSPATGRTPVFVSPTPPPPPPPLPSALSTSSLRASMTSTGSGSVPGSGSPPATALQAPAVGSGSAPLQ
      PRD[C]               EPGSGSPMHGAGDAKPIPTSISSATGLIENRPQSPATGRTPVFVSPTPGSGSPPLPSALSTSSLRASMTSTPPPPVPPPPPPPATALQAPAVGSGSAPLQ
      PRD[D]               EPGSGSPMHGAGDAKPIPTSISSATGLIENRPQSPATGRTPVFVSPTPGSGSPPLPSALSTSSLRASMTSTGSGSVPGSGSPPATALQAPAVPPPPAPLQ
      PRD[E]               EPGSGSPMHGAGDAKPIPTSISSATGLIENRPQSPATGRTPVFVSPTPGSGSPPLPSALSTSSLRASMTSTGSGSVPGSGSPPATALQAPAVGSGSAPLQ
      PRD[F]               EPGSGSPMHGAGDAKPIPTSISSATGLIENRPQSPATGRTPVFVSPTPGSGSPPLPSALSTSSLRASMTSTGSGSVPGSGSPPATALQAPAVGSGSAPLQ

                                      E                                    F
human WAVE1 377------------IAPGVLHPAPPPIAPPLVQPSPPVARAAPVCETVPVHPLPQGEVQGLPPPPPPPPLPPPGIRPSSPVTVTALAHPPSGLHPTPSTAPGPHVPLMPPSPPS-476
      PRD[null]            IAPGVLHPAGSGIAGSLVQPSPPVARAAPVSETVPVHPLPQGEVQGLPPGSGSPPLGSGGIRPSSPVTVTALAHPPSGLHPTPSTAPGPHVPLMPPSPPS
      PRD[A]               IAPGVLHPAGSGIAGSLVQPSPPVARAAPVSETVPVHPLPQGEVQGLPPGSGSPPLGSGGIRPSSPVTVTALAHPPSGLHPTPSTAPGPHVPLMPPSPPS
      PRD[B]               IAPGVLHPAGSGIAGSLVQPSPPVARAAPVSETVPVHPLPQGEVQGLPPGSGSPPLGSGGIRPSSPVTVTALAHPPSGLHPTPSTAPGPHVPLMPPSPPS
      PRD[C]               IAPGVLHPAGSGIAGSLVQPSPPVARAAPVSETVPVHPLPQGEVQGLPPGSGSPPLGSGGIRPSSPVTVTALAHPPSGLHPTPSTAPGPHVPLMPPSPPS
      PRD[D]               IAPGVLHPAGSGIAGSLVQPSPPVARAAPVSETVPVHPLPQGEVQGLPPGSGSPPLGSGGIRPSSPVTVTALAHPPSGLHPTPSTAPGPHVPLMPPSPPS
      PRD[E]               IAPGVLHPAPPPIAPPLVQPSPPVARAAPVSETVPVHPLPQGEVQGLPPGSGSPPLGSGGIRPSSPVTVTALAHPPSGLHPTPSTAPGPHVPLMPPSPPS
      PRD[F]               IAPGVLHPAGSGIAGSLVQPSPPVARAAPVSETVPVHPLPQGEVQGLPPPPPPPPLPPPGIRPSSPVTVTALAHPPSGLHPTPSTAPGPHVPLMPPSPPS

human WAVE1 477------------QVIPASEPKRHPSTLPVISDARSVLLEAIRKGIQLRKVEEQREQEAKHERIENDVATILSRRIAVEYSDSEDDSEFDEVDWLE-559
      PRD[null]            QVIPASEPKRHP
      PRD[A]               QVIPASEPKRHP
      PRD[B]               QVIPASEPKRHP
      PRD[C]               QVIPASEPKRHP
      PRD[D]               QVIPASEPKRHP
      PRD[E]               QVIPASEPKRHP
      PRD[F]               QVIPASEPKRHP
```

B

	WAVE1(PRD)	WAVE1 (PRD [null])	WAVE1 (PRD [B])
MW (Da), Theoretical	23,465	22,633	22,733
MW (Da), Measured	22,982	22,723	22,218
Percent Accuracy (100*Measured/Theoretical)	97.9	100.4	97.7
Reduced χ^2	0.529	0.714	0.472

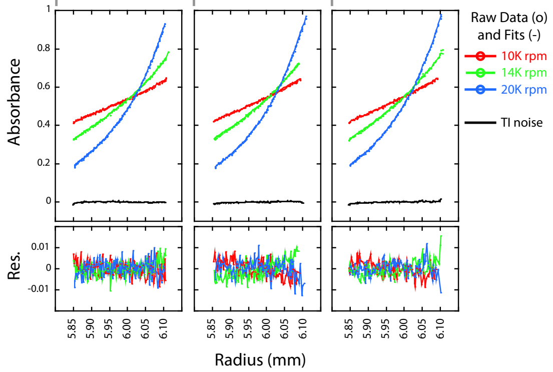

C

	WAVE1 (PRD [B]), 15.5 μM		
Pfn2 (μM)	91.2	44.8	21.7
MW(Da), Measured	43,292	39,779	33,794
Pfn2:WAVE1 (PRD [B])	1.4	1.17	0.77
Reduced χ^2	0.765	1.022	1.383

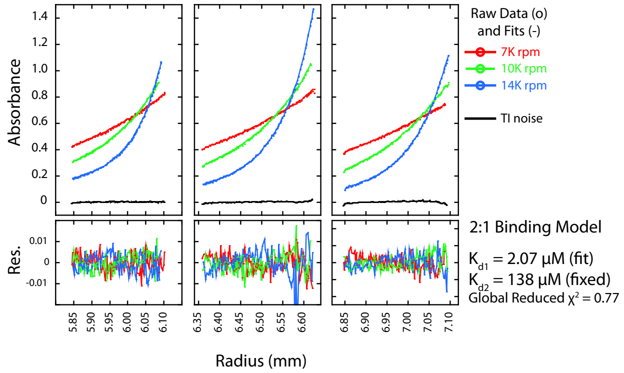

D

Construct	Profilin 2 Affinity [μM]	Profilin 2 Error [χ^2]	Profilin 1 Affinity [μM]	Profilin 1 Error [χ^2]
**PRD [null]**	**138**	**0.789**	**813**	**0.844**
**PRD [A]**	**51**	**1.103**	**703**	**0.797**
**PRD [B]**	**2**	**0.770**	**22**	**1.253**
**PRD [C]**	**12**	**0.690**	**251**	**0.795**
**PRD [D]**	**145**	**0.690**	**6577**	**0.657**
**PRD [E]**	**182**	**0.684**	**569**	**0.806**
**PRD [F]**	**4**	**1.104**	**68**	**0.937**

Figure EV4.

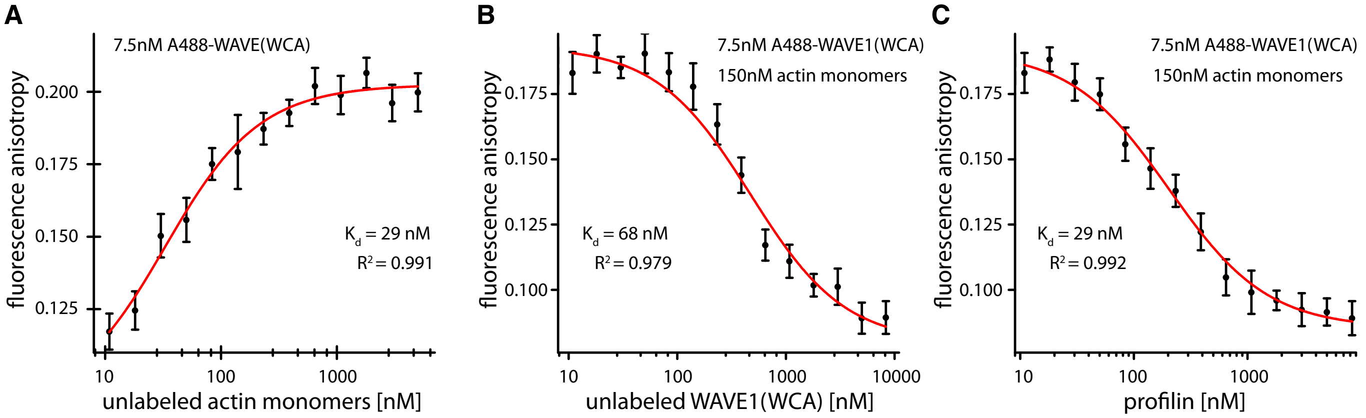

D

Interaction	Affinity [nM]	Figure	Method
**unlabed actin : unlabeled WAVE1 (WCA)**	**68 ± 13**	Suppl. Fig 7B	anisotropy (competition)
**unlabed actin : Alexa488-WAVE1 (WCA)**	**29 ± 3**	Suppl. Fig 7A	anisotropy
**Atto540Q-actin : Alexa488-WAVE1 (WCA)**	**61 ± 4**	Fig 5B	FRET
**Atto540Q-actin : Alexa488-WAVE1 (PWCA)**	**21 ± 1**	Fig 5B	FRET
**unlabed actin : unlabeled profilin**	**29 ± 4**	Suppl. Fig 7C	anisotropy (competition)
**Atto540Q-actin : unlabeled profilin**	**72 ± 11**	Fig 5C	FRET (competition)

Figure EV5. Summary of bimolecular equilibrium dissociation constants as determined from FRET- or fluorescence anisotropy-based measurements.

A Fluorescence anisotropy of 4 nM Alexa488-WAVE1 WCA as a function of increasing concentrations of Latrunculin B-stabilized monomeric actin. The data were fit using the quadratic solution to the 1:1 equilibrium binding equation (red line).

B Fluorescence anisotropy of 7.5 nM Alexa488-WAVE1 WCA in the presence of 175 nM Latrunculin B-stabilized monomeric actin as a function of increasing WAVE1 WCA concentrations. A kinetic competition model was fit to the data (red curve).

C Fluorescence anisotropy of 7.5 nM Alexa488-WAVE1 WCA in the presence of 175 nM Latrunculin B-stabilized monomeric actin as a function of increasing profilin concentrations. A kinetic competition model was fit to the data (red curve).

D Summary of affinities for the binary interactions determined from FRET- or fluorescence anisotropy-based measurements as indicated.

Data information: (A–C) Error bars are standard deviations from three independent measurements. (D) Errors are SEM.

 © 2017 The Authors
