# Supplementary material for: WH2 and proline‐rich domains of WASP‐family proteins collaborate to accelerate actin filament elongation
Source: EMBO J. 2017 Nov 15;37(1):102–21. doi: 10.15252/embj.201797039 (PMC5753033; doi:10.15252/embj.201797039)
Supplement: Supplementary file 2 — Movie EV1 [file EMBJ-37-102-s002.zip › Bieling_Movie_EV1_legend.docx]

**Movie EV 1. NPFs promote filament elongation locally.** TIRF microscopy of actin polymerization on a WAVE1-micropattern (magenta) in the presence of 1uM profilin-actin visualized by a filament-binding probe (5nM Alexa488-UTRN_N_, green).
